# The learning curve of transanal total mesorectal excision for rectal cancer is associated with local recurrence: results from a multicentre external audit

**DOI:** 10.1111/codi.15722

**Published:** 2021-06-09

**Authors:** Stefan E. Van Oostendorp, H. J. (Eric) Belgers, Jeroen C. Hol, Pascal G. Doornebosch, Eric J. Th. Belt, Steven J. Oosterling, Miranda Kusters, H. J. (Jaap) Bonjer, Colin Sietses, Jurriaan B. Tuynman, E. J. Boerma, D. Creemers, E. J. De Graaf, J. A. B. van der Hoeven, M. N. Sosef, H. B. A. C. Stockmann, E. P. van der Stok, R. C. L. M. Vuylsteke

**Affiliations:** ^1^ Department of Surgery Amsterdam UMC Vrije Universiteit Amsterdam Cancer Centre Amsterdam Amsterdam The Netherlands; ^2^ Department of Surgery Zuyderland Medical Center Heerlen The Netherlands; ^3^ Department of Surgery Gelderse Vallei Hospital Ede The Netherlands; ^4^ Department of Surgery IJsselland Hospital Capelle a/d Ijssel The Netherlands; ^5^ Department of Surgery Albert Schweitzer Hospital Dordrecht The Netherlands; ^6^ Department of Surgery Spaarne Gasthuis Hoofddorp The Netherlands

**Keywords:** TaTME, rectal cancer, local recurrence, learning, transanal, total mesorectal excision

## Abstract

**Aim:**

Transanal total mesorectal excision (TaTME) has been suggested as a potential solution for the resection of challenging mid and low rectal cancer. This relatively complex procedure has been implemented in many centres over the last years, despite the absence of long‐term safety data. Recently, concern has arisen because of an increase in local recurrence in the implementation phase. The aim of this study was to assess the correlation between accumulated experience and local recurrences.

**Method:**

An independent clinical researcher performed an external audit of consecutive series of all TaTME procedures in six centres in the Netherlands. Kaplan–Meier estimated local recurrence rates were calculated and multivariate Cox proportional hazards regression analysis performed to assess risk factors for local recurrence. Primary outcome was the local recurrence rate in the initial implementation (cases 1–10), continued adoption (cases 11‐40) and prolonged experience (case 41 onward).

**Results:**

Six hundred and twenty‐four consecutive patients underwent TaTME for rectal cancer with a median follow‐up of 27 months (range 1–82 months). The estimated 2‐ and 3‐year local recurrence rates were 4.6% and 6.6%, respectively. Cox proportional hazards regression revealed procedural experience to be an independent factor in multivariate analysis next to advanced stage (ycMRF+, pT3‐4, pN+) and pelvic sepsis. Corrected analysis projected the 3‐year local recurrence rates to be 9.7%, 3.3% and 3.5% for the implementation, continued adoption and prolonged experience cohorts, respectively.

**Conclusion:**

This multicentre study shows a high local recurrence rate (12.5%) after implementation of TaTME which lowers to an acceptable rate (3.4%) when experience increases. Therefore, intensified proctoring and further precautions must be implemented to reduce the unacceptably high risk of local recurrence at units starting this technique.

**What does this paper add to the literature?**

This study describes the results from six centres in the Netherlands. The audit shows that despite efforts at structured training and proctoring, the implementation phase of transanal total mesorectal excision (TaTME) was associated with an increased risk of local recurrence which improved with accumulated experience. This emphasizes the need to refine structured training programmes and extend the duration of proctoring, the importance of case selection and above all the absolute need for robust audited data from prospective trials to determine the role of TaTME in the treatment of rectal cancer.

​

## INTRODUCTION

Transanal total mesorectal excision (TaTME) was introduced to improve both clinical and long‐term outcomes for patients with low and mid rectal cancer [1]. Early adopters of the TaTME technique in high‐volume centres claimed promising clinicopathological results in selected TaTME cohorts compared with matched or historic cohorts of laparoscopic TME [2, 3]. These promising results provoked the interest of colorectal surgeons in using the TaTME technique for mid and distal rectal cancer. Nevertheless, the surgical community acknowledged that the technique is highly complex and requires training [4]. Subsequently it was considered that widespread implementation might have been premature pending robust data on reproducible long‐term outcomes [4, 5]. In particular, high‐quality evidence regarding long‐term outcomes after TaTME is still missing.

In‐depth analysis to quantify the learning curve by means of a cumulative sum (CUSUM) method has identified that an unsupervised ‘autodidact’ learning curve with the primary outcome of morbidity constitutes approximately 40 cases in centres with extensive experience in both single‐port and minimally invasive surgery [6, 7]. In another CUSUM‐based analysis of anastomotic leakage risk a tipping point was identified at 50 cases [8]. Interestingly, Persiani et al. found two cut‐off points: an initial reduction in both operation time and major complications was seen after 54 cases, and a further decrease in major complications at 69 cases and operating time at 87 cases [9]. In addition, specific intraoperative complications such as urethral injury in male patients and systemic carbon dioxide emboli have been collectively reported by early adopters and seem to relate to an unfamiliar bottom‐up approach to the pelvic anatomy with risk of entering a wrong plane and different technical aspects, such as the continuous high‐flow insufflation in a confined space [10, 11]. This indicates that TaTME is a substantially different surgical concept rather than a modification of approach or instruments, and has created awareness of the potential hazards of widespread adoption. Therefore, multiple nations have initiated structured training pathways in order to safely implement the technique in new centres [12, 13, 14, 15, 16]. These programmes consist of detailed study of the anatomy, observation of live surgery, cadaver training and, ideally, on‐site proctoring. Proctorship by an experienced surgeon aims to prevent intraoperative mistakes and improve surgical technique, which ought to limit exposure of patients to hazardous and long learning curves for individual surgeons [4, 17].

Despite these unprecedented implementation measures, a concerning local recurrence rate of 10% in the first series of 10 patients in 12 Dutch centres occurred in a structured training programme [18]. In addition, the Norwegian colorectal cancer group declared a moratorium on TaTME following a nationwide audit which revealed an estimated local recurrence rate of 11.6% at 2.4 years [19]. Interestingly, a majority of the local recurrences in both studies showed a multifocal pattern, which led to speculation about the potential presence of technical or executional issues [18, 19]. In contrast to the aforementioned studies, multiple respectably sized cohorts of TaTME procedures with a median follow‐up of approximately 2 years showed that good local recurrence rates, ranging from 2% to 6%, can be achieved in dedicated centres [8, 20, 21, 22, 23, 24].

The present audit study aimed to assess the local recurrence rate during the initial implementation, continued adoption and prolonged experience of TaTME in six hospitals in the Netherlands.

## METHOD

The primary endpoint was local recurrence rate in relation to surgical experience and the secondary endpoint was anastomotic take down and end colostomy rate in restorative procedures in relation to surgical experience.

An external audit of the full electronic patient records of a prospectively tracked series of all consecutive TaTME procedures was performed in six high‐volume hospitals (one started 2012, one in 2013, one in 2014, two in 2015 and one in 2016) including all the original imaging reports, operation notes and pathology reports. Preoperative work‐up and follow‐up were performed according to the national guidelines. In summary, this constitutes a full colonoscopy with biopsy of the lesion, MRI of the rectum, carcinoembryonic antigen (CEA) and imaging of the liver and thorax by CT scan or ultrasound and x‐ray, respectively. Neoadjuvant long‐course chemoradiotherapy was given in case of threatened margin to the mesorectal fascia (MRF) or cN2 disease. For frail patients, short‐course radiotherapy with a long interval to surgery was considered as an alternative option. Short‐course 5 × 5 Gy neoadjuvant radiotherapy has been given for those with clinical T3 disease with more than 5 mm extramural invasion and/or cN1 disease. Follow‐up was according to the national guidelines, which recommend 6‐monthly imaging of chest and liver and CEA during the first 2 years and thereafter yearly up to 5 years [25].

The cumulative local recurrence rate was estimated by the Kaplan–Meier method and inter‐group difference was assessed by log‐rank test. A separate subgroup analysis was performed for patients in whom initial or restage MRI after neoadjuvant therapy if applicable showed no threatened margin to the MRF. For comparative analysis of increasing institutional experience, case sequence numbers were categorized into initial implementation (cases 1–10), continued adoption (cases 11–40) and prolonged experience (case 41 onward). Cut‐off values were established in advance based on the first 10 to make a comparison with the previous report of the Dutch structured training pathway and the second cut‐off at 40 based on previous evaluation of the learning curve [6, 18]. To identify risk factors for local recurrence, the effects of covariates were analysed using a univariate Cox proportional hazards regression model. Covariates with an effect of *p* < 0.10 were subsequently entered into a multivariable Cox proportional hazards regression model in which a *p*‐value of <0.05 was considered significant.

## RESULTS

A total of 624 patients who underwent TaTME for rectal cancer entered this cohort with a median follow‐up of 27 months (range 1–82 months). All consecutive cases of TaTME for primary rectal cancer since the start of this technique in each of the six centres were included; the date of surgery ranged from March 2012 to May 2020. The caseload among the six participating centres ranged between 47 and 227. The three cohorts defined as the initial implementation (cases 1–10), continued adoption (cases 11–40) and prolonged experience (case 41 onward) constituted 60, 180 and 384 patients, respectively.

### Baseline

The majority of included patients were men (73.7%) and 19.4% of the study population was classified as obese [body mass index (BMI) ≥30 kg/m^2^]. Almost half of all tumours (46.3%) were located below or within 3 cm of the anorectal junction (ARJ). Clinical tumour staging showed cT4 in 6.0% and cT3 in 66.8%. The MRF was threatened in a quarter of the cohort (*n* = 154, 24.7%) of which less than half (*n* = 68, 10.9%) showed a persistent threatened margin to the MRF upon restaging after neoadjuvant treatment. Synchronous distant metastases were present in 47 patients (7.5%); these were mostly hepatic followed by a pulmonary location (Table 1).

**TABLE 1:** Patient characteristics (*N* = 624)

Characteristic		*n* or value
Sex, *n* (%)	Male	440 (70.5%)
	Female	184 (29.5%)
BMI (kg/m^2^), mean ± SD		26.7 ± 4.2
Age (years), mean ± SD		66.0 ± 10.9
ASA classification, *n* (%)	I	106 (17.0%)
	II	401 (64.3%)
	III	116 (18.6%)
	IV	1 (0.2%)
Height from ARJ (cm), mean ± SD		3.7 ± 2.7
Clinical tumour stage (cT), *n* (%)	cTis/TVA hgr	3 (0.5%)
	cT1	21 (3.4%)
	cT2	145 (23.3%)
	cT3	415 (66.8%)
	cT4	37 (6.0%)
	Missing	3 (–)
Clinical nodal stage (cN), *n* (%)	N0	297 (48.1%)
	N1	186 (30.1%)
	N2	135 (21.8%)
	Missing	6 (–)
Synchronous metastasis (cM), *n* (%)	No	577 (92.5%)
	Yes	47 (7.5%)
MRF threatened, *n* (%)	Pre‐neoadjuvant (c‐) RT	154 (24.7%)
	Persistent upon restaging	68 (10.9%)
Preoperative therapy, *n* (%)	None	220 (35.3%)
	5 × 5 short interval	137 (22.0%)
	5 × 5 long interval	76 (12.2%)
	Chemoradiotherapy	190 (30.4)
	Systemic chemotherapy	1 (0.2%)

Abbreviations: ARJ, anorectal junction; ASA, American Society of Anesthesiologists; BMI, body mass index; CRT, chemoradiotherapy; MRF, mesorectal fascia; RT, radiotherapy; SD, standard deviation; TVA hgr, tubulovillous adenoma with high‐grade dysplasia.

### Operative details

A low anterior TME resection was performed in 539 patients (86.4%) in this cohort. In these a primary anastomosis was constructed without diversion in 103 (16.5%), anastomosis with a diverting ileostomy in 337 (54.0%) and nonrestorative end‐colostomy (Hartmann) in 99 patients (15.9%). An intersphincteric resection with creation of an end‐colostomy was performed in 80 patients (12.5%) and a TaTME resection as part of a proctocolectomy was done in five patients. Intraoperative complications are listed in Table 2 and comprised 1 urethral injury, 5 carbon dioxide emboli, 11 cases of pelvic bleeding, 14 documented purse‐string failures and 21 intraoperative rectal perforations.

**TABLE 2:** Operation details (*N* = 624)

		*n* (%)
Procedure	LAR	103 (16.5%
	LAR – ileostomy	337 (54.0%)
	LAR – colostomy	99 (15.9%)
	ISR – colostomy	80 (12.8%)
	Proctocolectomy	5 (0.8%)
Anastomosis	Not performed	180 (29.0%)
	Stapled	378 (60.9%)
	Hand‐sewn	63 (10.1%)
	Missing	3 (–)
Conversion	No conversion	595 (95.4%)
	Laparotomy	15 (2.4%)
	Pfannenstiel	5 (0.8%)
	Laparoscopy	7 (1.1%)
	Open APR	1 (0.2%)
Extraction site	Transanal	204 (34.8%)
	Pfannenstiel	271 (46.2%)
	(Contralateral) McBurney	33 (5.6%)
	Umbilical trocar site	15 (2.6%)
	Laparotomy	13 (2.2%)
	Stoma site	42 (7.2%)
	Missing	38 (–)
Intraoperative complications	Urethral injury	1 (0.2%)
	CO_2_ embolus	5 (0.8%)
	Pelvic bleeding	11 (1.8%)
	Visceral injury	7 (1.1%)
	Purse‐string failure	14 (2.2%)
	Rectal perforation	21 (3.4%)
	Anastomotic problem €	62 (10.0%)
	Technical problem transanal phase	3 (0.5%)

Abbreviations: APR, abdominoperineal resection; ISR, intersphincteric resection; LAR, low anterior resection.

### Postoperative morbidity

The overall postoperative morbidity rate was 53.7%; this was further classified according to Clavien–Dindo grades as shown in Table 3. Short‐term anastomotic leakage and/or pelvic abscess occurred in approximately one out of five of both restorative and non‐restorative procedures. Anastomotic takedown and creation of an end‐colostomy due to septic complications occurred in 42 out of 443 (9.5%) restorative procedures. The anastomotic takedown rate following septic anastomotic complications decreased from 13.5% in the first 25 restorative TaTME procedures to 11.5% in the second and 7.6% in the third 25, and to 2.2% in procedures 76–100 (*p =* 0.023).

**TABLE 3:** Morbidity (*N* = 624)

		*n* (%)
Postoperative complications (30 day)	None – CD 0	289 (46.3)
	CD I	57 (9.1%)
	CD II	120 (19.2%)
	CD IIIa	24 (3.8%)
	CD IIIb	93 (14.9%)
	CD IV	36 (5.8%)
	CD V	5 (0.8%)
Major surgical morbidity (30 day)	CD ≥III	149 (23.9%)
Short‐term leakage or abscess (30 day)	Anastomosis (*N* = 443)	89 (20.1%)
	Non‐restorative (*N* = 181)	31 (17.1%)
Overall pelvic sepsis^a^		140 (22.4%)
Anastomotic takedown^b^ (*N* = 443)		42 (9.5%)

Abbreviation: CD, Clavien–Dindo.

^a^ Includes early and late complications (leakages, abscess and/or sinus).

^b^ Unintended take down of anastomosis and creation of end colostomy due to septic complications.

### Pathology

An involved circumferential margin was observed in 20 cases (3.2%) and a positive distal margin in 4 (0.6%). Major defects of the specimen were reported in 19 cases (3.1%; Table 4).

**TABLE 4:** Pathology (*N* = 624)

		*n* (%)
Pathological T‐stage	(y)pT0	63 (10.1%)
	(y)pT1	66 (10.6%)
	(y)pT2	248 (39.8%)
	(y)pT3	242 (38.8%)
	(y)pT4	4 (0.6%)
	Missing	1 (–)
Quality of specimen (Quirke)	No defects	539 (87.2%)
	Minor defects	60 (9.7%)
	Major defects	19 (3.1%)
	Not reported	6 (–)
CRM involvement (≤1 mm)^a^		20 (3.2%)
DRM involvement (≤1 mm)		4 (0.6%)
Nodal stage	pN0	446 (71.5%)
	pN1	138 (22.1%)
	pN2	40 (6.4%)
Nodes harvested (mean ± SD)^b^		16.9 ± 7.6

Abbreviations: CRM, circumferential resection margin; DRM, distal resection margin; SD, standard deviation.

^a^ One missing.

^b^ Excluding five proctocolectomies.

### Primary outcome: local recurrence

**FIGURE 1:**
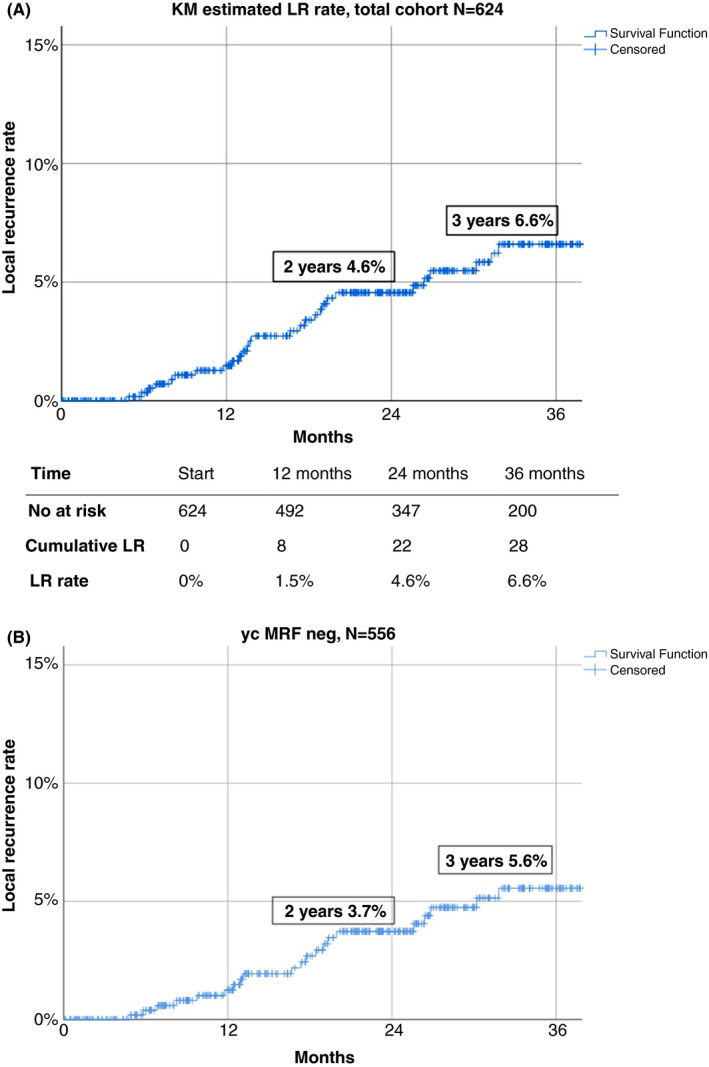
(A) Kaplan–Meier (KM) estimated local recurrence (LR) rate, total cohort (*n* = 624), (B) Kaplan–Meier (KM) estimated local recurrence (LR) rate, subgroup of non‐threatened margin to the mesorectal fascia (*n*=556)

**FIGURE 2:**
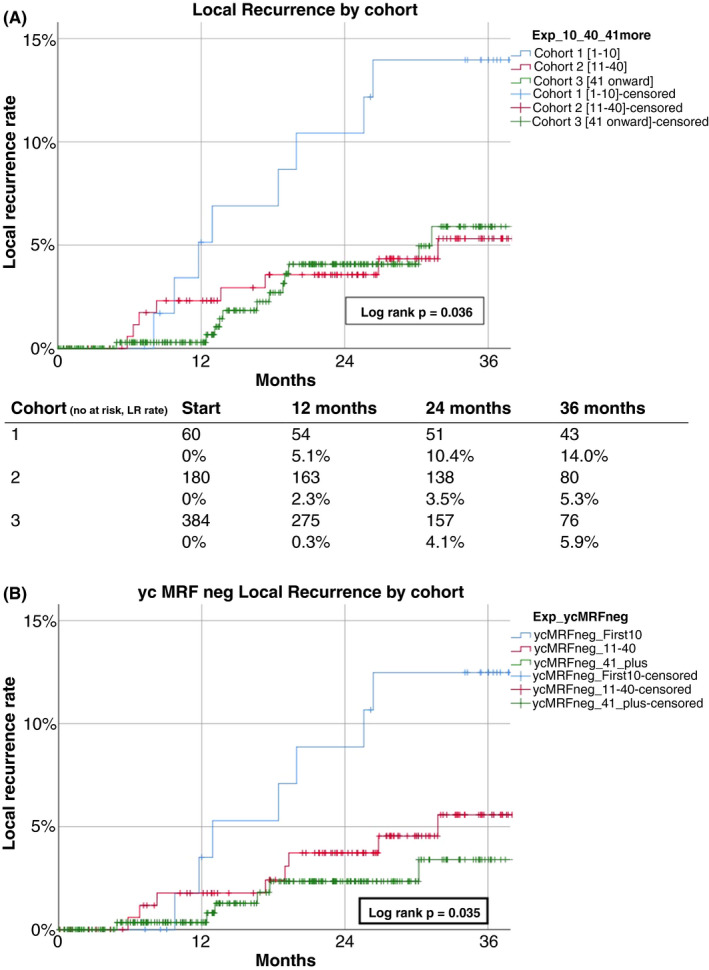
(A) Local recurrence rate by experience of total cohort (*N *= 624) (cohort 1 cases 1–10, cohort 2 cases 11–40, cohort 3 case 41 onwards). (B) Local recurrence rate by experience, subgroup of non‐threatened margin to the mesorectal fascia (*n *= 566). Cohorts as in (A). Log rank test for comparative analysis

Thirty patients developed a local recurrence (4.8%) after a median interval of 17 months (range 5–61 months) from index surgery. The predominant location was presacral (*n* = 16; 53%) while a multifocal pattern was observed in six local recurrences (20%; Table 5) Kaplan–Meier survival analysis showed an estimated local recurrence rate in the total study population of 4.6% at 2 years and 6.6% at 3 years (Figure 1A). Comparative analyses of the three predefined cohorts showed a 3‐year local recurrence rate of 14.0% in the initial implementation, 5.3% during continued adoption and 5.9% with prolonged experience (*p *= 0.036) (Figure 2A). Exclusion of patients with a persistent threatened margin after neoadjuvant therapy showed a Kaplan–Meier estimated local recurrence rate of 3.7% at 2 years and 5.6% at 3 years (see Figures 1B and 2B).

**TABLE 5:** Follow‐up (*N* = 624)

Follow‐up (months)	Mean ± SD)	29.0 ± 18.3
	Median (range)	26.8 (1–82)
Local recurrence, *n* (%)	Overall	30 (4.8%)
Interval to local recurrence (months)	Median (range)	17 (5–61)
Location of local recurrence^a^, *n* (%)	Presacral	16 (53.3%)
	Anterior	1 (3.3%)
	Lateral	2 (6.7%)
	Anastomosis	3 (10.0%)
	Rectal stump	2 (6.7%)
	Multifocal	6 (20.0%)

Abbreviations: SD, standard deviation.

^a^ Denominator is total local recurrence (*N* = 30).

**FIGURE 3:**
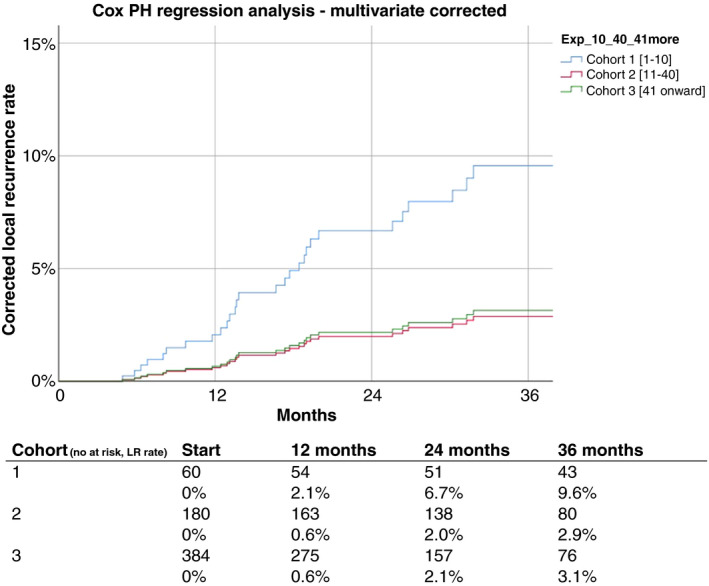
Corrected Cox proportional hazards (PH)multivariate regression analysis. Corrected for variables significant (*p* < 0.05) in multivariate analysis (Table 6)

Cox proportional hazard regression analysis to identify predictive risk factors for local recurrence revealed experience to be a consistent independent predicting factor in uni‐ and multivariate analysis next to a persistent threatened margin to the MRF following neoadjuvant therapy, advanced stage pT3‐4, presence of pathological lymph nodes and pelvic sepsis. (Table 6). Adjusted Cox regression analysis to correct for case mix projected the 3‐year local recurrence rate to be 9.6%, 2.9% and 3.1% for the three cohorts, respectively. Both the continued adoption phase [hazard ratio (HR) 0.290, 95% CI 0.108–0.780, *p* = 0.014] and prolonged experience (HR 0.318, 95% CI 0.127–0.795, *p* = 0.014) had a significant lower hazard of developing a local recurrence compared with the initial implementation cohort (Figure 3, Table 6).

**TABLE 6:** Cox proportional hazards regression analysis

		Univariate				Multivariate		
		Event/total	HR	95% CI	*p*‐value	HR	95% CI	*p*‐value
Experience	Cases 1–10	9/60	*1.0* (ref.)			*1.0* (ref.)		
	Cases 11–40	8/180	0.339	0.130–0.881	**0.026**	0.290	0.108–0.780	**0.014**
	Case 41 onward	13/384	0.394	0.165–0.939	**0.035**	0.318	0.127–0.795	**0.014**
Sex	Female	9/184	*1.0* (ref.)			NA		
	Male	21/440	0.978	0.448–2.136	0.955	NA		
BMI (kg/m^2^)	<30	24/503	*1.0* (ref.)			NA		
	≥30	6/121	0.976	0.399–2.389	0.958	NA		
Height from ARJ (cm)	>3	12/335	*1.0* (ref.)			NA		
	≤3	18 /289	1.802	0.868–3.742	0.114	NA		
Previous local excision	No	29/569	*1.0* (ref.)			NA		
	Yes	1/55	0.397	0.054–2.914	0.363	NA		
Clinical M1	No	27/577	*1.0* (ref.)			NA		
	Yes	3/47	2.151	0.649–7.126	0.210	NA		
Chemoradiotherapy	No	15/434	*1.0* (ref.)			*1.0* (ref.)		
	Yes	15/190	2.471	1.205–5.066	**0.014**	1.894	0.854–4.201	0.116
Post‐CRT MRF+	No	23/556	*1.0* (ref.)			*1.0* (ref.)		
	Yes	7/68	2.990	1.274–7.017	**0.023**	2.774	1.055–7.299	**0.039**
(y)pT‐stage	0–2	8/378	*1.0* (ref.)			*1.0* (ref.)		
	3–4	22/246	4.668	2.077–10.490	**<0.001**	2.562	1.077–6.092	**0.033**
CRM involved (<1 mm)	No	27/603	*1.0* (ref.)			*1.0* (ref.)		
	Yes	3/20	5.073	1.529–16.828	**0.008**	2.020	0.561–7.276	0.282
DRM involved	No	30/617	*1.0* (ref.)			NA		
	Yes	0/4	0.049	0.000–2.7 × 10^7^	0.740	NA		
pN stage	Negative	12/447	*1.0* (ref.)			*1.0* (ref.)		
	Positive	18/177	4.213	2.027–8.756	**<0.001**	3.127	1.447–6.759	**0.004**
IO defect^a^	No	25/532	*1.0* (ref.)			NA		
	Yes	5/89	1.595	0.607–4.188	0.344	NA		
Pelvic sepsis	No	18/488	*1.0* (ref.)			*1.0* (ref.)		
	Yes	12/140	2.147	1.033–4.464	**0.041**	2.530	1.169–5.472	**0.018**

Abbreviations: ARJ, anorectal junction; BMI, body mass index; Clinical M1, synchronous distant metastasis; CRM, circumferential resection margin; CRT, chemoradiotherapy; DRM, distal resection margin; HR, hazard ratio; MRF, mesorectal fascia; NA, not applicable.

Significant in univariate analysis if *p* < 0.010 and significant in Multivariate analysis significant if *p* < 0.050.

^a^ IO defect: composite of either intraoperative rectal perforation, purse‐string failure or defects of the anastomosis, pelvic sepsis, composite of short and long‐term anastomotic leakage, pelvic abscess or presacral sinus.

Pelvic sepsis and an unintended intraoperative connection between the rectal lumen and pelvic cavity (purse‐string failure, rectal perforation or anastomotic defect) were additionally assessed as potential risk factors. Twelve local recurrences occurred in patients with pelvic sepsis (12 out of 140, 8.6%) versus 18 in patients without pelvic sepsis (18 out of 484, 3.7%). Five local recurrences occurred after an unintended open connection (5 out of 86, 5.8%) versus 25 local recurrences without a connection (25 out of 535, 4.7%). In uni‐ and multivariate Cox regression, pelvic sepsis was related to an increased risk of local recurrence (HR 2.530, 95% CI 1.159–5.472, *p* = 0.018) while an open connection did not show a significantly increased risk for the development of local recurrence.

## DISCUSSION

This external audit of a prospective multicentre consecutive cohort of TaTME procedures (*N* = 624) shows that the incidence of local recurrence following TaTME for rectal cancer is associated in multivariate analysis with surgical experience in addition to advanced pT‐ and pN‐stage and pelvic sepsis. A relatively high rate of LR in the initial implementation phase was observed which diminished to a low percentage during further implementation in the six centres. These results show that the learning curve is partially responsible for the increased risk of local recurrences for the TaTME procedure. For cases without a threatened margin, the local recurrence rate for the first 10 procedures was 13% but below 5% for the following series (Figure 2B). This learning curve effect was also visualized for conversion (10%, 6% and 3% for the three groups, respectively) and for anastomotic takedown due to septic complications (Supplementary Material). Centres currently planning or starting with TaTME should be cautious, and adequate training, patient selection and case volume seem very relevant for obtaining safe results.

After the declared moratorium on TaTME in Norway various renowned centres for minimally invasive rectal cancer surgery have published (multi‐)institutional cohort studies with a 2%–6% crude local recurrence [8, 20, 21, 22, 23, 24]. In response to the audit of the Dutch structured training pathway revealing a crude 10% local recurrence rate in the first 10 consecutive patients, Warrier et al. reported a 2% local recurrence rate among 300 patients at a minimum of 2 years follow‐up within the Australasian structured training pathway for TaTME [26]. In‐depth analysis of the organization of the Australasian and UK implementation pathway might show particular differences in entry criteria, training, case selection, technique and competency sign‐off which could offer insights into the diverging oncological results [13, 15, 26]. The structured training pathway in the Netherlands is currently on hold and will need further refinement and more strict governance upon its restart [12]. In addition to annual volume requirements and an extended duration of proctoring, continued quality assurance by video assessment and repeated external audit of clinical outcomes might be beneficial [27, 28].

The introduction of TaTME (implementation) has been transparently studied and evaluated by a global collaborative, with unprecedented public sharing (i.e. data and videos at conferences) of early unfavourable outcomes in order to improve the technique. Moreover, extensive training and other precautions, which have tried to adhere to the IDEAL framework, have nevertheless failed to prevent the current setback and scepticism about the oncological safety of the technique [29]. The current detailed findings of TaTME‐associated local recurrences in the start of the learning curve should be compared with laparoscopic and robotic‐assisted TME resection, of which the long‐term data on local recurrence during the implementation phase are not well registered.

The expected benefits in especially difficult low rectal cancer cases have tempted participating centres to select challenging cases even early in the learning curve. From the implementation cohort (the first 10 cases in each centre), 15 out of 60 (25%) patients would not have met the eligibility criteria (cT4 or cT3 ≤2 mm to the MRF, previous local excision or synchronous metastasis) of the current benchmark for laparoscopic TME surgery, namely the COLOR 2 trial​ (Supplementary Material) [30]. Unfortunately, in the early phase of TaTME patients with low tumours, a narrow pelvis and threatened margins were offered this novel technique, which would currently be highly disputed since the learning curve should not incorporate such difficult cases [31]. Moreover, included cases were often more advanced in terms of difficulty compared with selected cohorts as seen in the ALaCaRT, ACOSOG Z6051 and COLOR II trials since the participating centres have become referral centres for patients in pursuit of a restorative or sphincter‐saving procedure [30, 32, 33]. Nevertheless, patients should be fully informed and consent to undergo any surgical procedure, and especially a new surgical technique including potential unknown hazards and uncertain long‐term outcomes [34]. The potential negative effects are mostly present in difficult cases: a small pelvis, high BMI, anterior or low situated tumours. For mid rectal cancer, an immediate bailout when encountering any difficulties can be made by converting to the standard technique, laparoscopic abdominal TME, and it is recommended to do this with a low threshold.

For optimal assessment of the local recurrence rate, adequate follow‐up for a minimum of 3 years for an entire cohort is desirable; this is not yet available. Given the current debate on the safety of TaTME with respect to (multifocal) local recurrence postponing the publication of our current results was considered unethical. Multiple groups have assessed the learning curve by CUSUM analysis to be around 40–50 procedures [6, 7, 8, 9, 35]. Therefore, the chosen cut‐off of 40 cases, next to the first cut‐off at 10 procedures to serve as reference from the previous audit of 12 centres was considered appropriate. A learning curve is generally measured by CUSUM analysis rather than a case ranking method including an arbitrary cut‐off to define subgroups as performed in this study. However, such analysis requires an extensive cohort, ideally of a single surgeon. Another limitation is that the current study did not assess the volume effect, i.e. cases per time unit, on (long‐term) outcome since we focused on institutional rather than individual surgeon experience.

When introducing new techniques, a thorough and well‐designed scientific evaluation according to the IDEAL framework is essential to guarantee patient safety [34]. Equipoise towards an intervention should be based on reliable data which the surgical community should prove using registries and clinical trials with a high standard of data quality. Clinical trials with quality assurance are ongoing but it must be acknowledged that the adoption of TaTME without proper audit might have gone too fast [36].

## CONCLUSION

TaTME is a complex procedure with a learning curve that not only affects short‐term morbidity but is also associated with an increased risk of local recurrence; however, this improves both in terms of lower morbidity and local recurrence rates with greater experience.

## COLLABORATORS

E. J. Boerma (Department of Surgery, Zuyderland Medical Center, Heerlen, The Netherlands), D. Creemers (Department of Surgery, Zuyderland Medical Center, Heerlen, The Netherlands), E. J. De Graaf (Department of Surgery, IJsselland Hospital, Capelle a/d Ijssel, The Netherlands), J. A. B. van der Hoeven (Department of Surgery, Albert Schweitzer Hospital, Dordrecht, The Netherlands), M. N. Sosef (Department of Surgery, Zuyderland Medical Center, Heerlen, The Netherlands), H. B. A. C. Stockmann (Department of Surgery, Spaarne Gasthuis, Hoofddorp, The Netherlands), E. P. van der Stok (Department of Surgery, IJsselland Hospital, Capelle a/d Ijssel, The Netherlands), R. C. L. M. Vuylsteke (Department of Surgery, Spaarne Gasthuis, Hoofddorp, The Netherlands).

## CONFLICT OF INTEREST

All authors declare nothing to disclose.

## AUTHOR CONTRIBUTIONS

All authors have participated in the conceptualization of the study, SvO and JH acquired data, SVO, MK and JT performed the data analysis. All authors participated in the interpretation of the data. SVO, HJB and JT drafted the article and all authors revised it critically. All authors approved the final version to be published.

## DATA AVAILABILITY STATEMENT

Individual patient data are not available due to patient privacy protection as agreed in data transfer agreement between the participating centres. However, the congregated data in this study are available upon reasonable request from the corresponding author.

## ETHICAL APPROVAL

This study was approved by the ethics committee of the Amsterdam UMC, location VUmc. No additional informed consent was required for this study.

## Supporting information

Supplementary Material
