# Supplementary material for: The learning curve of transanal total mesorectal excision for rectal cancer is associated with local recurrence: results from a multicentre external audit
Source: Colorectal Dis. 2021 Jun 9;23(8):2020–9. doi: 10.1111/codi.15722 (PMC8453958; doi:10.1111/codi.15722)
Supplement: Supplementary file 1 — Supplementary Material [file CODI-23-2020-s001.docx]

# Supplementary material

## Figure S1 – effect of experience on anastomotic takedown rate

## Table S1 – effect of experience on anastomotic takedown rate

| **P = 0.023** | 1st 25 | 2nd 25 | 3rd 25 | 4th 25 |
| --- | --- | --- | --- | --- |
| Anastomosis preserved | 128 | 100 | 73 | 45 |
|  | 86,5% | 88,5% | 92,4% | 97,8% |
| Takedown &  end-colostomy | 20 | 13 | 6 | 1 |
|  | 13,5% | 11,5% | 7,6% | 2,2% |

## Table S2 – Groups baseline and outcome

|  |  | **Total cohort** | **Case #1-10** | **Case #11-40** | **Case #41** | **P value** | **Statistical Test** |
| --- | --- | --- | --- | --- | --- | --- | --- |
|  |  | **N = 624** | **n=60** | **n= 180** | **N = 384** |  |  |
| **Median FU in months** |  | **26.8** | **47.3** | **33.8** | **21.3** |  |  |
| Sex | Female | 184 (29.5) | 10 (16.7) | 64 (35.6) | 110 (28.6) | **0.018** | **Chi square** |
|  | Male | 440 (70.5) | 50 (83.3) | 116 (64.4) | 274 (71.4) |  |  |
| BMI | < 30 | 503 (80.6) | 48 (80.0) | 137 (76.1) | 318 (82.8) | 0.172 | Chi square |
|  | ≥ 30 | 121 (19.4) | 12 (20.0) | 43 (23.9) | 66 (17.2) |  |  |
| Height | >3 cm from ARJ | 335 (53.7) | 29 (48.3) | 80 (44.4) | 226 (58.9) | **0.004** | **Chi square** |
|  | ≤3 cm from ARJ | 289 (46.3) | 31 (51.7) | 100 (55.6) | 158 (41.1) |  |  |
| Clinical Tumor stage (cT) | cT0 - Tis | 3 (0.5) | 0 (0) | 0 (0) | 3 (0.8) | 0.405 | Fishers exact |
|  | *cT1* | 21 (3.4) | 2 (3.3) | 6 (3.4) | 13 (3.4) |  |  |
|  | *cT2* | 145 (23.3) | 11 (18.3) | 51 (28.8) | 83 (21.6) |  |  |
|  | *cT3* | 415 (66.8) | 45 (75.0) | 113 (63.8) | 257 (66.9) |  |  |
|  | *cT4* | 37 (6.0) | 2 (3.3) | 7 (4.0) | 28 (7.3) |  |  |
| Clinical Nodal stage (cN) | *N0* | 297 (48.1) | 29 (48.3) | 83 (46.9) | 185 (48.6) | 0.986 | Chi square |
|  | *N1* | 186 (30.1) | 17 (28.3) | 56 (31.6) | 113 (29.7) |  |  |
|  | *N2* | 135 (21.8) | 14 (23.3) | 38 (21.5) | 83 (21.8) |  |  |
| Clinical M stage | No | 577 (92.5) | 57 ( 95.0) | 172 (95.6) | 348 (90.6) | 0.100 | Fishers exact |
|  | Yes | 47 (7.5) | 3 (5.0) | 8 (4.4) | 36 (9.4) |  |  |
| Initial MRI MRF+ | no | 470 (75.3) | 51 (85.0) | 145 (80.6) | 274 (71.4) | **0.011** | **Chi square** |
|  | yes | 154 (24.7) | 9 (15.0) | 35 (19.4) | 110 (28.6) |  |  |
| Persisting MRF+ after (Chemo)RTX | no | 556 (89.1) | 58 (96.7) | 164 (91.1) | 334 (87.0) | **0.043** | **Fishers exact** |
|  | yes | 68 (10.9) | 2 (3.3) | 16 (8.9) | 50 (13.0) |  |  |
| Chemoradiotherapy | no | 434 (69.6) | 42 (70.0) | 120 (66.7) | 272 (70.8) | 0.604 | Chi square |
|  | yes | 190 (30.4) | 18 (30.0) | 60 (33.3) | 112 (29.2) |  |  |
| Restorative procedure | No | 181 (29.0) | 14 (23.3) | 61 (33.9) | 106 (27.6) | 0.185 | Chi square |
|  | Yes | 443 (71.0) | 46 (76.7) | 119 (66.1) | 278 (72.4) |  |  |
| Conversion | No | 593 (95.5) | 54 (90.0) | 169 (93.9) | 370 (97.1) | **0.022** | **Fishers exact** |
|  | Yes | 28 (4.5) | 6 (10.0) | 11 (6.1) | 11 (2.9) |  |  |
| (y)pT-stage | 0-2 | 378 (60.6) | 32 (53.3) | 120 (77.7) | 226 (58.9) | 0.102 | Chi square |
|  | 3-4 | 236 (39.4) | 28 (46.7) | 60 (33.3) | 158 (41.1) |  |  |
| (y)pN-stage | negative | 447 (71.6) | 42 (70.) | 130 (72.2) | 275 (71.6) | 0.954 | Chi square |
|  | positive | 177 (28.4) | 18 (30.0) | 50 (27.8) | 109 (28.4) |  |  |
| CRM involved | no | 603 (96.8) | 58 (96.7) | 174 (96.7) | 371 (96.9) | 0.999 | Fishers exact |
|  | yes | 20 (3.2) | 2 (3.3) | 6 (3.3) | 12 (3.1) |  |  |
| Quality - major defects | no | 599 (96.9) | 56 (100) | 170 (94.4) | 373 (97.6) | 0.067 | Fishers exact |
|  | yes | 19 (3.1) | 0 (0) | 10 (5.6) | 9 (2.4) |  |  |
| Postoperative moribidty | No | 301 (48.3) | 31 (51.7) | 89 (49.4) | 181 (47.3) | 0.761 | Chi square |
|  | Yes | 322 (51.7) | 29 (48.3) | 91 (50.6) | 202 (52.7) |  |  |
| Major Surgical moribidy | No | 475 (76.1) | 44 (73.3) | 134 (74.4) | 297 (77.3) | 0.652 | Chi square |
|  | Yes | 149 (23.9) | 16 (26.7) | 46 (25.6) | 87 (22.7) |  |  |
| Pelvic Sepsis | No | 484 (77.6) | 44 (73.3) | 135 (75.0) | 305 (79.4) | 0.366 | Chi square |
|  | yes | 140 (22.4) | 16 (26.7) | 45 (25.0) | 79 (20.6) |  |  |
| Anastomotic failure | no | 401 (90.5) | 39 (84.8) | 102 (85.7) | 260 (93.5) | 0.020 | Chi square |
|  | yes | 42 (9.5) | 7 (15.2) | 17 (14.3) | 18 (6.5) |  |  |
